# Comprehensive quantitative magnetic resonance imaging assessment of skeletal muscle pathophysiology in golden retriever muscular dystrophy: Insights from multicomponent water T2 and extracellular volume fraction

**DOI:** 10.1002/nbm.5278

**Published:** 2024-10-21

**Authors:** Ericky Caldas de Almeida Araujo, Inès Barthélémy, Yves Fromes, Pierre‐Yves Baudin, Stéphane Blot, Harmen Reyngoudt, Benjamin Marty

**Affiliations:** ^1^ NMR Laboratory, Neuromuscular Investigation Center Institute of Myology Paris France; ^2^ Université Paris Est Créteil, INSERM, IMRB, Créteil, France; EnvA, IMRB Maisons‐Alfort France

**Keywords:** multicomponent T2, muscular dystrophy, pathophysiology, quantitative MRI, specificity, water T2

## Abstract

Quantitative MRI and MRS have become important tools for the assessment and management of patients with neuromuscular disorders (NMDs). Despite significant progress, there is a need for new objective measures with improved specificity to the underlying pathophysiological alteration. This would enhance our ability to characterize disease evolution and improve therapeutic development. In this study, qMRI methods that are commonly used in clinical studies involving NMDs, like water T2 (T2_H2O_) and T1 and fat‐fraction (FF) mapping, were employed to evaluate disease activity and progression in the skeletal muscle of golden retriever muscular dystrophy (GRMD) dogs. Additionally, extracellular volume (ECV) fraction and single‐voxel bicomponent water T2 relaxometry were included as potential markers of specific histopathological changes within the tissue. Apart from FF, which was not significantly different between GRMD and control dogs and showed no trend with age, T2_H2O_, T1, ECV, and the relative fraction of the long‐T2 component, A_2_, were significantly elevated in GRMD dogs across all age ranges. Moreover, longitudinal assessment starting at 2 months of age revealed significant decreases in T2_H2O_, T1, ECV, A_2_, and the T2 of the shorter‐T2 component, T2_1_, in both control and GRMD dogs during their first year of life. Notably, insights from ECV and bicomponent water T2 indicate that (I) the elevated T2_H2O_ and T1 values observed in dystrophic muscle are primarily driven by an expansion of the extracellular space, likely driven by the edematous component of inflammatory responses to tissue injury and (II) the significant decrease of T2_H2O_ and T1 with age in control and GRMD dogs reflects primarily the progressive increase in fiber diameter and protein content during tissue development. Our study underscores the potential of multicomponent water T2 relaxometry and ECV to provide valuable insights into muscle pathology in NMDs.

## Graphical abstract

In this retrospective study, on top of fat‐fraction, water T2 (T2_H2O_) and native T1, we assessed the extracellular volume (ECV) fraction and the multi‐exponential behavior of water T2 relaxation in the skeletal muscle of golden retriever muscular dystrophy (GRMD) dogs. Our findings demonstrate the sensitivity of the explored metrics to dystrophic changes and skeletal muscle growth and maturation. Insights from ECV and bicomponent water T2 relaxometry suggest extracellular edema as the primary driver of increased T2_H2O_ and native T1 in GRMD.

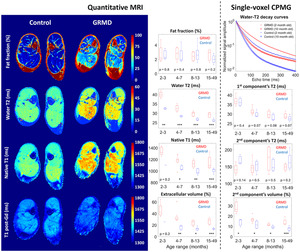

**Abbreviations**
BMD: Becker muscular dystrophy
BW/px: Bandwidth per pixel
CPMG: Carr–Purcell–Meiboom–Gill
DMD: Duchenne muscular dystrophy
DNA: Deoxyribonucleic acid
ECG: Electrocardiogram
ECV: Extracellular volume fraction
EPG: Extended phase graph
FA: Flip angle
FF: Fat fraction
Gd: Gadolinium
GRAPPA: Generalized autocalibrating partially parallel acquisitions
GRMD: Golden retriever muscular dystrophy
HCT: Hematocrit
ISIS: Image‐selected in vivo spectroscopy
qMRI: Quantitative MRI
qMRI/S: Quantitative MRI/MRS
MSME: Multi‐slice multi‐spin‐echo
NMD: Neuromuscular disorder
ROI: Region of interest
TC: Tibialis cranialis
TI: Inversion time
T1_H2O_
: Water T1
T2_H2O_
: Water T2

## 1 INTRODUCTION

Duchenne muscular dystrophy (DMD) is a severe degenerative neuromuscular disorder (NMD), caused by X‐linked inherited mutations that limit the production of dystrophin, which is a critical structural protein in the muscle fibers. The lack of dystrophin results in chronic tissue injury, characterized by repeated cycles of necrosis, regeneration and concurrent inflammation. Over time, this leads to progressive muscle fibrosis, waste and replacement by fat. Symptoms typically appear in the first few years of life, indicated by delayed motor milestones, and evolve rapidly, with loss of ambulation around the age of 12. As the disease progresses, cardiac and respiratory impairments result in premature death around the age of 25.^1^

Golden retriever muscular dystrophy (GRMD) is a genetically determined model of the human DMD disease, sharing similar histopathological alterations and clinical symptoms. Most preclinical trials on GRMD focus on the first year of the disease, which closely parallels the first 10 to 15 years of DMD. During this period, dogs exhibit early signs of muscle weakness, followed by severe progression indicated by postural changes and significantly impaired locomotion. Subsequently, locomotor signs tend to stabilize, and dogs can generally live through adulthood, although respiratory and cardiac impairments end up leading to premature death around 6–7 years of age.^2^

Quantitative MRI/MRS (qMRI/S) has become an important tool for the assessment and management of patients with NMDs.^3^, ^4^, ^5^, ^6^, ^7^, ^8^ For instance, fat‐fraction (FF) maps quantify the degree of muscle‐fat replacement, allowing to precisely evaluate the stage and rate of the disease progression, which is valuable information for assessing the disease heterogeneity and for optimizing treatment strategies. On the other hand, water T2 (T2_H2O_) and water‐T1 (T1_H2O_) maps reveal the presence of pathophysiological alterations that precede muscle wasting.^4^, ^7^, ^9^, ^10^ In particular, T2_H2O_ has been shown to be abnormally increased in DMD^8^, ^11^ and to provide some predictive value concerning future muscle‐fat replacement^4^, ^6^, ^7^ and loss of function,^5^ in different NMDs. However, different from FF, which quantifies a specific morphological change within the tissue, T2_H2O_ and T1_H2O_ are not specific, being increased in the presence of different histopathological tissue alterations that result in augmented muscle water content and free water fraction, such as cell swelling, interstitial edema, and inflammation.^11^, ^12^, ^13^, ^14^ This lack of specificity has hindered their qualification as biomarkers.

One way to increase specificity would be to assess the histological tissue water compartmentation. A particularly interesting qMRI method for assessing tissue water compartmentation is the extracellular volume (ECV) fraction, obtained using T1 maps acquired before and after intravenous gadolinium (Gd)‐based contrast agent injection. In cardiac applications, ECV has been shown to reveal the extent of focal and diffuse myocardial fibrosis in muscular dystrophy patients^15^, ^16^, as well as other pathological alterations resulting in expanded extracellular space such as cardiac amyloidosis and myocarditis.^17^ Recently, ECV has been shown to correlate with skeletal muscle fibrosis in a fibrotic mice model.^18^ In Becker muscular dystrophy (BMD), a milder form of dystrophinopathy, where fibrosis is an important phenotypic feature, ECV was abnormally increased even in normal appearing muscles with normal T2_H2O_.^19^ Another interesting and promising approach, relatively unexplored in the realm of muscle MRI, involves investigating the actual multiexponential behavior of the T2 relaxation of the water signal in skeletal muscle. When properly measured and analyzed, the T2 relaxation of muscle water is characterized by a distribution of T2 values, instead of a single T2 obtained from a monoexponential fitting, as used for standard T2_H2O_ mapping.^20^ There is now strong evidence indicating that this behavior reflects primarily the tissue water compartmentation at the histological level.^13^, ^14^, ^21^, ^22^ More recently, in a cohort of patients, bicomponent water T2 was more sensitive and specific in detecting the presence of disease than the conventional monoexponential T2, allowing, for instance, the differentiation of myopathies.^11^

In this study, we investigated changes in tissue water compartmentation in the skeletal muscle of GRMD dogs using multicomponent water T2 and ECV measurements, in addition to conventional FF, T2_H2O_, and T1 mapping techniques. We hypothesize that these complementary methods may provide further insights into the pathophysiological alterations underlying the characteristic changes in T2_H2O_ and T1 observed in dystrophic skeletal muscle.

## 2 MATERIALS AND METHODS

### 2.1 Animals

This is a retrospective study comprising 17 GRMD and 7 age‐matched control (healthy golden retriever) dogs, as summarized in Table 1. Among these, eight GRMD and four control dogs had multiple (ranging between two and six) visits, with dogs' ages throughout the visits varying between 2 and 24 months. The age of the dogs that underwent a single visit ranged from 2 to 49 months. In total, 55 dog examinations were conducted, comprising 30 in GRMD (6 in 2‐ to 3‐month‐old, 12 in 4‐ to 7‐month‐old, 5 in 8‐ to 13‐month‐old, and 7 in 15‐ to 49‐month‐old dogs) and 25 in control dogs (3 in 2‐ to 3‐month‐old, 4 in 4‐ to 7‐month‐old, 7 in 8‐ to 13‐month‐old, and 11 in 15‐ to 49‐month‐old dogs). All GRMD dogs were diagnosed through DNA analysis as previously described.^23^

**TABLE 1:** Detailed description of the study cohort, indicating the number of dogs that underwent single or multiple visits and the age at each visit.

Age group			1	2		3		4		
Dogs' age (months)			2	4–5	6–7	8–10	12–13	15–19	24–28	36–49
Single visits	Nb of dogs per age	GRMD	1		2			2		4
		Control						1		2
Dogs with longitudinal follow‐up	GRMD	Dog 1		X	X					
		Dog 2		X	X	X				
		Dog 3	X		X					
		Dog 4	X		X					
		Dog 5	X		X					
		Dog 6			X	X	X			
		Dog 7	X		X		X			
		Dog 8	X		X		X	X		
	Control	Dog 1	X		X	X	X	X	X	
		Dog 2	X		X	X	X	X	X	
		Dog 3	X		X	X	X	X	X	
		Dog 4			X		X	X	X	

*Note*: X marks indicate the time points at which each dog was examined in the longitudinal studies.

Abbreviation: GRMD, golden retriever muscular dystrophy.

The study was conducted in conformity with the European regulatory laws concerning the use of animals for research and the French Ministry of research (Ministère de l'Enseignement Supérieur et de la Recherche) under the following approval numbers: #3678, #23763, #36911, and 20/12/12‐18.

### 2.2 Data acquisition and analysis

Blood samples were acquired from the dogs before MRI acquisition for assessing hematocrit (HCT). MRI and MRS data acquisition was conducted using a 3‐T whole‐body clinical system (Magnetom PRISMA^fit^, Siemens Healthineers, Erlangen, Germany). Throughout the entire examination, dogs were under isoflurane anesthesia and placed on a heating pad, while their cardiac and respiratory rates were continuously monitored. A 15‐channel birdcage transceiver coil was used for RF transmission and signal detection at the level of the distal pelvic limbs. Quantitative fat‐water MRI was performed using a multi‐echo gradient‐echo sequence (3D acquisition, TEs = 2.75 and 3.95, TR = 10 ms, FA = 3°, resolution = 0.5 × 0.5 × 1.0 mm^3^, 104 slices, BW/px = 490 Hz), and FF maps were reconstructed based on a 2‐pt Dixon method. T2_H2O_ maps were generated by fitting an extended phase graph (EPG)‐based signal model to multi‐slice multi‐spin‐echo (MSME) data (inter‐echo spacing = 9.6 ms, Nb of echoes = 17, nominal excitation/refocusing FA = 90°/180°, TR = 3 s, resolution = 1.4 × 1.4 × 5 mm^3^, 11 slices, slice gap = 12.5 mm, BW/px = 445 Hz, partial Fourier = 6/8, GRAPPA^24^ acceleration factor = 2, acquisition time = 3 min 32 s), as described in Marty et al.^25^ Maps of T1 were generated from single inversion recovery Look‐Locker data (2D FLASH, 1400 radial spokes, TE/echo spacing = 2.1/3.42 ms, TR = 10 s, TI = 30 ms, FA = 5°, BW/px = 820 Hz, resolution = 1.1 × 1.1 × 8 mm^3^, slice gap = 12 mm, 5 slices, acquisition time = 50 s) using a dictionary matching approach, as described in Marty et al.^26^ Muscle T1 maps were obtained at two timepoints, before (native T1) and 15 min after an intravenous injection of a gadolinium‐based contrast agent (Dotarem [0.2 mmol/kg], Guerbet) (T1 post‐Gd).

For assessing ECV, the blood T1 was measured in the left ventricular cavity before and 10 minutes after contrast‐agent injection. This was necessary since the spatial resolution of the T1 maps of the pelvic limbs was not sufficient for allowing precise assessment of blood T1 in one of the major arteries. For cardiac acquisitions, the body coil was used for RF transmission and an 18‐channel flexible‐phased array coil in combination with a 32‐channel spine coil for signal reception. Cardiac T1 maps were generated from data acquired using the standard ECG‐triggered modified Look‐Locker inversion recovery sequence^27^ (Single‐slice True FISP, TE/echo‐spacing = 1.19/2.7 ms, TR = 550 ms, TI = 260 ms, Inter‐echo spacing = 2.7 ms, FA = 35°, BW/px = 930 Hz, resolution = 1.9 × 1.9 × 5.5 mm^3^, partial Fourier = 6/8, phase res. = 80%, acquisition time = 22 s).

Multicomponent water T2 was assessed in the right tibialis cranialis (TC) muscle using a fat‐suppressed (CHESS^28^) single‐voxel (~1 × 1 × 4 cm^3^) image‐selected in‐vivo spectroscopy Carr–Purcell–Meiboom–Gill (ISIS‐CPMG) sequence^13^ (inter‐echo‐spacing = 2 ms, echo‐train‐length = 200, TR = 7.5 s, 2‐step phase cycle). The TC muscle was chosen for its geometry that simplifies the single‐voxel positioning, contributing to the methodological standardization between acquisitions. Only the even echoes were kept, and each water T2 decay curve was normalized by the signal amplitude at TE = 4 ms. The following bi‐exponential model was fitted to the normalized water T2 decay curves using nonlinear least squares:

(1) $$S\left(\mathrm{TE}\right)=a_{1}e^{-\mathrm{TE}/T2_{1}}+a_{2}e^{-\mathrm{TE}/T2_{2}}$$

The T2 values of the first and second components, T2_1_ and T2_2_, along with the second component's relative fraction, $A_{2}=a_{2}/\left(a_{1}+a_{2}\right)$, were computed for each examination.

With the goal to confront qMRI and ISIS‐CPMG data, ROIs were drawn within the right TC muscle on the out‐of‐phase Dixon images, the T1‐weighted images, and the T2‐weighted images. For the T2_H2O_ and native‐T1 maps, the five central slices were selected for the analysis, except for FF, where the slices were selected to coincide with the selected slice positions on the other parametric maps. For each dataset, the mean value of all the pixels within the five ROIs was calculated for each parameter map.

From the mean muscle native T1 and T1 post‐Gd, the blood native T1 and T1 post‐Gd, and the hematocrit, the mean ECV in the right TC muscle was calculated as follows:

(2) $$\mathrm{ECV}=\frac{∆R1_{\text{muscle}}}{∆R1_{\text{blood}}}\left(1-\mathrm{HCT}\right)$$

Where, $∆R1=\frac{1}{T1_{\text{postGd}}}-\frac{1}{T1_{\text{native}}}$

All data processing was conducted in Python 3.11 and MATLAB R2022b.

### 2.3 Statistics

We investigated the relationship between the assessed qMRI/S metrics and age during the dogs' first year of life in GRMD and control groups by fitting separate linear mixed‐effects models for each group. In these models, age was included as a fixed effect, and individual dogs were treated as a random effect for the intercept to account for repeated measures within subjects. To assess differences between GRMD and control dogs, we used the Mann–Whitney *U*‐test at four distinct age ranges. The null hypothesis was rejected for every *p*‐value less than 0.05. All the statistical analysis was conducted in MATLAB R2022b.

## 3 RESULTS

### 3.1 Quantitative MRI

**FIGURE 1:**
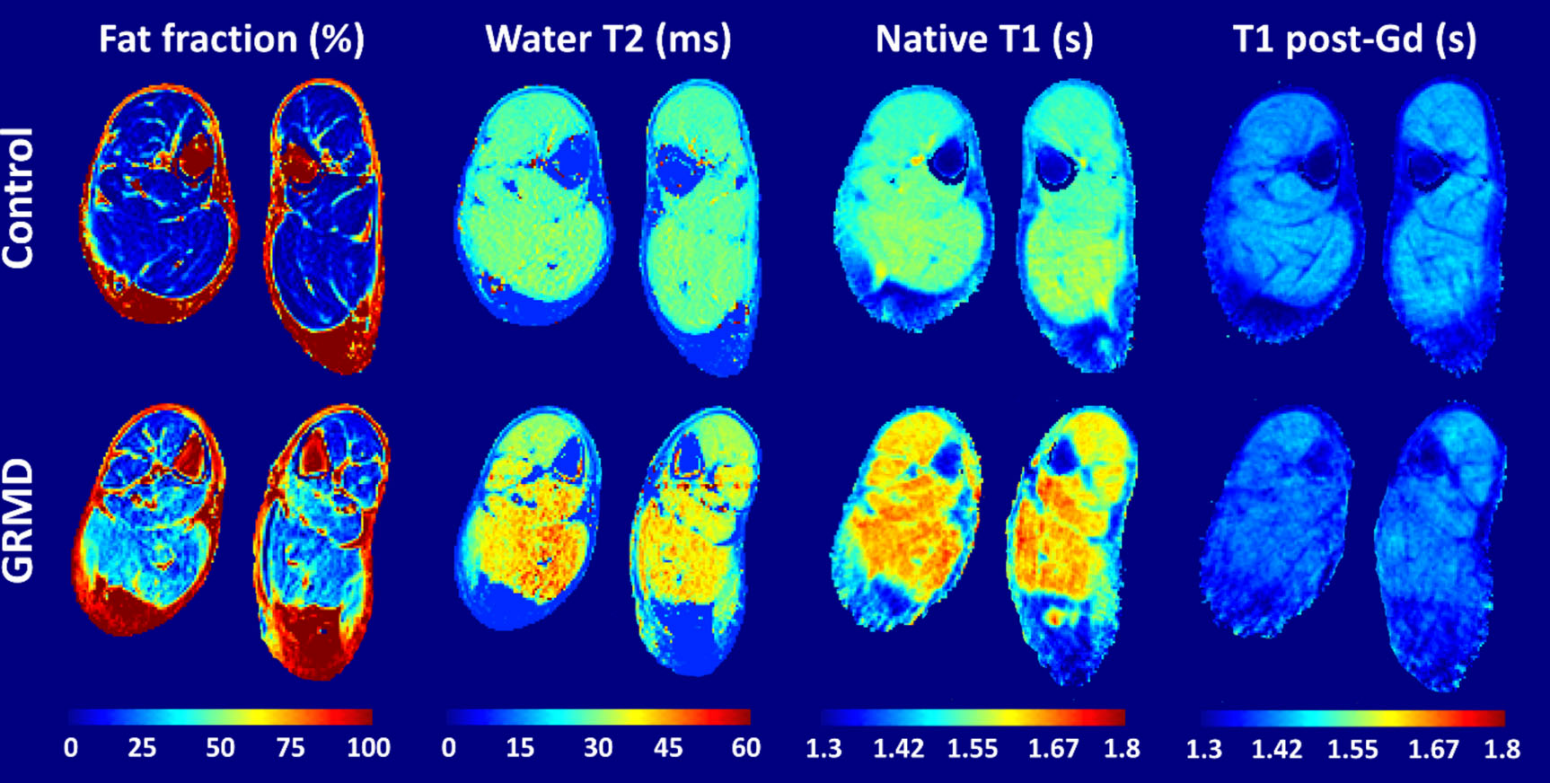
Examples of axial fat‐fraction (FF), water T2 (T2_H2O_), native T1 and T1 postgadolinium maps obtained at the level of the distal pelvic limbs in a control and a GRMD dog. GRMD, golden retriever muscular dystrophy.

**FIGURE 2:**
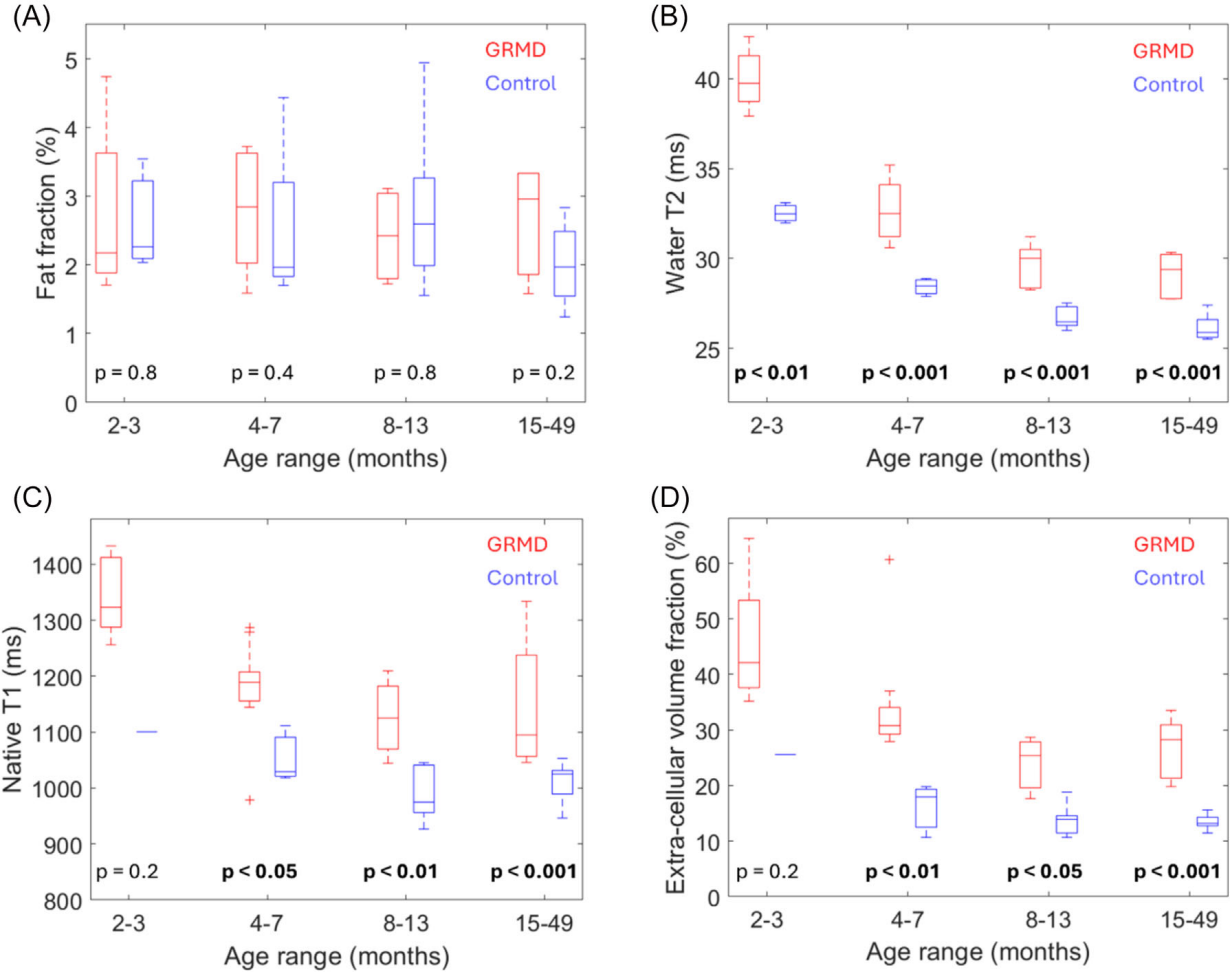
Boxplots showing the distribution of the mean fat‐fraction (A), water T2 (B), native T1 (C), and extracellular volume fraction (D) observed in the tibialis cranialis muscle of GRMD and control dogs at four age ranges. The *p*‐values comparing GRMD and control dogs in each age range are indicated. GRMD, golden retriever muscular dystrophy.

Figure 1 shows examples of the qMRI data obtained in a control and a GRMD dog. Due to incomplete examinations in some dogs/visits, only 48 and 43 out of the 55 examinations included T1 mapping and ECV measurement, respectively. In Figure 2, the boxplots show the distribution of the mean FF, T2_H2O_, native T1 and ECV values characterizing the TC muscle across four age ranges (2–3, 4–7, 8–13, and 15–49 months) for both GRMD and control dogs, where the *p*‐value comparing GRMD and control dogs within each age range is indicated. The corresponding medians and interquartile ranges are presented in Table 2. While no significant difference in FF was observed between GRMD and control dogs, T2_H2O_, native T1 and ECV were elevated in GRMD. However, due to a lack of control data for native T1 and ECV in young control dogs, the differences in these markers did not reach significance at the youngest age range (2–3 months). Notably, there is a visible progressive decrease in the median values of all three metrics with increasing age in both groups. This trend is more pronounced in the GRMD dogs, where they start higher and decrease more steeply compared to the control dogs. This observation is supported by the linear mixed‐effects model fits, with results summarized in Table 3. The model was characterized with a significant negative slope for all three metrics, with significantly different intercepts and slopes between GRMD and control dogs, except for the slope on the native T1.

**TABLE 2:** Summary of the results for all the qMRI/S metrics evaluated in each group of dogs at four age ranges, presented as median (interquartile range), alongside *p*‐values from the corresponding Mann–Whitney *U*‐tests.

Age range (months)		FF (%)	T2_H2O_ (ms)	T1 (ms)	ECV (%)	T2_1_ (ms)	T2_2_ (ms)	A_2_ (%)
2–3	GRMD	2.2 (1.7)	**39.7 (2.6)**	1323 (125)	42 (16)	36.4 (2.8)	141 (22)	**26.4 (9.5)**
	Control	2.3 (1.1)	**32.5 (0.8)**	1101	26	36.0 (3.7)	162 (21)	**16.3 (5.5)**
	*p*‐value	0.8	**<0.01**	0.2	0.2	0.4	0.14	**<0.05**
4–7	GRMD	2.8 (1.6)	**32.5 (2.9)**	**1189 (52)**	**30.8 (4.8)**	30.8 (1.9)	146 (22)	**16.5 (2.4)**
	Control	2.0 (1.4)	**28.5 (0.8)**	**1029 (70)**	**18.0 (6.9)**	29.0 (2.0)	156 (27)	**11.4 (7.1)**
	*p*‐value	0.4	**<0.001**	**<0.05**	**<0.01**	0.07	0.5	**<0.05**
8–13	GRMD	2.4 (1.2)	**30.0 (2.2)**	**1125 (113)**	**25.4 (8.3)**	29.6 (3.5)	178 (69)	**11.2 (1.7)**
	Control	2.6 (1.3)	**26.5 (1.0)**	**975 (85)**	**13.9 (3.1)**	27.1 (2.8)	147 (33)	**8.9 (2.0)**
	*p*‐value	0.8	**<0.001**	**<0.01**	**<0.05**	0.08	0.5	**<0.05**
15–49	GRMD	3.0 (1.5)	**29.4 (2.5)**	**1095 (181)**	**28.3 (9.6)**	28.6 (2.3)	149 (18)	**12.3 (3.9)**
	Control	2.0 (0.9)	**25.9 (1.0)**	**1025 (42)**	**13.2 (1.5)**	27.5 (1.9)	162 (38)	**8.2 (2.6)**
	*p*‐value	0.2	**<0.001**	**<0.001**	**<0.001**	0.07	0.2	**<0.001**

*Note*: T1: Native muscle T1. T2_H2O_: Water T2. T2_1_: first component's T2 (ISIS‐CPMG). T2_2_: second component's T2 (ISIS‐CPMG). A_2_: second component's volume fraction (ISIS‐CPMG). Results presented in bold correspond to statistically significant differences between GRMD and control groups, as confirmed by the corresponding *p*‐values.

Abbreviations: ECV, extracellular volume fraction; FF, fat‐fraction; GRMD, golden retriever muscular dystrophy.

**TABLE 3:** Summary of linear mixed‐effects model fittings of the explored qMRI/MRS metrics against age (from 2 to 15 months)—intercepts, slopes, and respective *p*‐values comparing GRMD and control dogs.

		FF (%)	T2_H2O_ (ms)	T1 (ms)	ECV (%)	T2_1_ (ms)	T2_2_ (ms)	A_2_ (%)
Intercept	GRMD	3.0 (1.1)	**40.8 (1.7)**	**1347 (71)**	**48.8 (8.5)**	38.0 (1.9)	139 (24)	**31.1 (3.8)**
	Control	2.5 (1.1)	**32.7 (1.1)**	**1175 (48)**	**23.8 (5.3)**	36.5 (2.7)	167 (20)	**15.3 (3.1)**
	*p*‐value	0.5	**<0.001**	**<0.001**	**<0.001**	0.4	0.07	**<0.001**
Slope	GRMD	−0.03 (0.17)^a^	**−1.1 (0.2)**	−22.3 (10.2)	**−2.3 (1.3)**	−0.9 (0.3)	2.1 (3.5)^a^	**−2.0 (0.6)**
	Control	0.03 (0.11)^a^	**−0.5 (0.1)**	−14.6 (1.9)	**−0.9 (0.5)**	−0.8 (0.3)	−1.4 (2.3)^a^	**−0.5 (0.3)**
	*p*‐value	0.6	**<0.001**	0.15	**<0.05**	0.6	0.1	**<0.001**

*Note*: T1: Native muscle T1. T2_H2O_: Water T2. T2_1_: first component's T2 (ISIS‐CPMG). T2_2_: second component's T2 (ISIS‐CPMG). A_2_: second component's volume fraction (ISIS‐CPMG). Results presented in bold correspond to statistically significant differences between GRMD and control groups, as confirmed by the corresponding *p*‐values.

Abbreviations: ECV, extracellular volume fraction; FF, at fraction; GRMD, golden retriever muscular dystrophy.

^a^ Not significantly different from zero.

### 3.2 Single‐voxel multicomponent water T2

**FIGURE 3:**
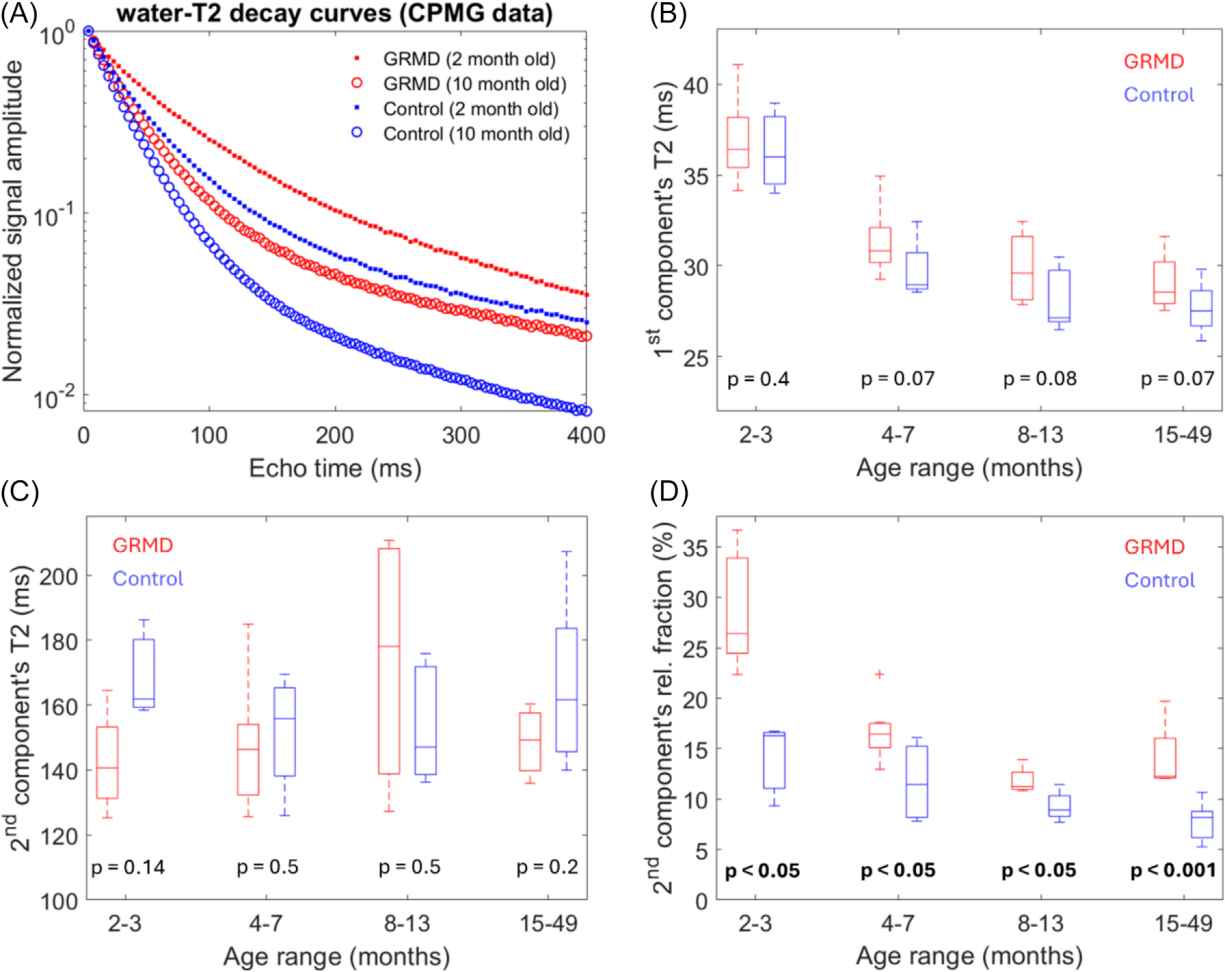
In (A), are displayed examples of normalized water T2 decay curves measured in the tibialis cranialis muscle of a GRMD and a control dog at two different timepoints (2 and 10 months old). The curves clearly deviate from a straight line, demonstrating their nonmonoexponential behavior. In (B)–(D), the boxplots illustrate the distribution of the first component's T2, the second component's T2, and its corresponding relative fraction, respectively, obtained from the bi‐exponential fitting of the water T2 decay curves. The *p*‐values comparing GRMD and control dogs in each age range are indicated. GRMD, golden retriever muscular dystrophy.

Due to incomplete examinations in some dogs/visits, only 45 out of the 55 examinations included single‐voxel CPMG acquisitions. Figure 3A shows examples of water T2 decay curves measured in the TC muscle of a GRMD and a control dog acquired at different timepoints (2 and 10 months old), where one may clearly notice their nonmonoexponential behavior and appreciate the impact of both age and disease on the water T2 relaxation. The water T2 decay curves were accurately modeled (see Equation (1)), resulting in a median mean square residual of 6.3 × 10^−6^ with an interquartile range of 5.0 × 10^−6^. In Figure 3B–D, the boxplots show the distribution of the bicomponent water T2 metrics, T2_1_ and T2_2_ and A_2_, across four age ranges (2–3, 4–7, 8–13, and 15–49 months) for both GRMD and control dogs, where the *p*‐value comparing GRMD and control dogs within each age range is indicated. The corresponding medians and interquartile ranges are presented in Table 2. While no significant differences between GRMD and control dogs were observed in the first and second components T2 values, the relative fraction of the long‐T2 component, A_2_ (Figure 3D), was significantly elevated in GRMD at all four age ranges. Notably, there is a visible progressive decrease in the median values of T2_1_ and A_2_ with increasing age in both groups. While trend in T2_1_ is similar between GRMD and control dogs, the trend in A_2_ is clearly more pronounced in the GRMD dogs, where they start higher and decrease more steeply compared to the control dogs. This observation is supported by the linear mixed‐effects model fits, with results summarized in Table 3. The model was characterized with significant negative slopes for both T2_1_ and A_2_, with significantly different intercept and slope between GRMD and control dogs on the A_2_ metric.

### 3.3 Supplementary material—Longitudinal histopathological data

Figure S1 presents longitudinal histological assessment of the TC muscle in a separate cohort of GRMD and control dogs, evaluated at 2, 6, and 12 months of age (unpublished data). Quantitative histopathological analysis was performed according to a previously described method.^29^ The data reveal an evident increase in myofiber diameter during muscle development, accompanied by a corresponding decrease in ECV fraction. Quantitative analysis further indicates that (I) the volume fraction of endomysial and perimysial connective tissue tends to decrease over time in both control and GRMD dogs, while being consistently elevated in GRMD; (II) adipose deposition barely develops in GRMD TC muscle, except in a few severely affected dogs at the age of 6 months; and (III) mononuclear cell infiltrates are mainly observed at early timepoints (2 and 6 months) declining by 12 months.

## 4 DISCUSSION

In this study, qMRI methods that are commonly used in clinical studies involving NMDs were employed to evaluate disease activity (T2_H2O_ and native T1) and progression (FF) in the skeletal muscle of GRMD dogs. Additionally, ECV and single‐voxel bicomponent water T2 relaxometry were included as potential markers of specific histopathological changes within the tissue.

As expected from the typical phenotype seen in GRMD^30^, ^31^, ^32^ (Figure S1), FF was not significantly higher than in controls, contrasting with what is observed in DMD patients, who experience a severe progression of muscle‐fat replacement in the distal leg muscles over time.^8^, ^33^ Previous studies have demonstrated the sensitivity of T2_H2O_ and native T1 to dystrophic tissue alterations in GRMD, and skeletal muscle growth in control and GRMD dogs.^30^, ^31^ Our results confirm these findings further, showing that the qMRI metrics T2_H2O_, native T1, and ECV, along with the bicomponent water T2 relaxometry metric A_2_, were consistently elevated in GMRD dogs across all age ranges. Moreover, longitudinal assessment starting at 2 months of age revealed significant decreases in T2_H2O_, native T1, ECV, T2_1_, and A_2_ in both control and GRMD dogs during their first year of life, confirming their sensitivity to muscle growth and maturation in both groups. These results are encouraging and suggest that potential positive effects of treatments can be assessed through these metrics, if the threshold values for normal tissue are established for different age ranges. Previous studies have demonstrated that various MRI metrics respond to gene therapy in GRMD dogs.^34^, ^35^ In clinical studies, T2_H2O_ was shown to significantly decrease in corticosteroid‐treated DMD patients^36^ and in sirolimus‐treated inclusion body myositis patients.^37^

Previous research on healthy skeletal muscle suggests that the first (A_1_ and T2_1_) and second (A_2_ and T2_2_) components characterizing the T2 relaxation of water represent the parenchymal and vascular compartments, respectively.^13^, ^22^ From this perspective, our CPMG data suggest an elevated relative vascular volume in the GRMD dogs, as indicated by the increased A_2_ values. This finding aligns with the elevated ECV values observed in the same dogs. Interestingly, while T2_1_, A_2_, and ECV were all sensitive to tissue growth and maturation, T2_1_, unlike A_2_ and ECV, was not significantly different between control and GRMD dogs and presented a very similar evolution with age in both groups. This suggests that these metrics are likely sensitive to distinct changes in diseased and developing muscle tissue, with the tissue alterations related to muscle growth and maturation, impacting all of them, and the dystrophic‐induced changes, impacting primarily A_2_ and ECV. It is worth noting that these findings are consistent with the parenchymal‐vascular compartmentation model. Muscle tissue growth and maturation are characterized by cellular hypertrophy^38^ (see Figure S1) and an increase in intracellular protein content.^39^ While the former translates into an augmented cellular volume fraction, which explains the progressive decrease observed in A_2_ and ECV as the dogs grow, the latter is expected to reduce the intracellular T2, accounting for the progressive decrease observed in T2_1_ during the same period. In contrast, inflammatory responses to acute and chronic tissue injury are anticipated exclusively in GRMD, commencing at early ages and diminishing during the first year of the disease.^2^, ^31^ This aligns with the elevated A_2_ and ECV values observed in GRMD dogs across all age ranges and the significantly faster decrease of both metrics in GRMD dogs with age.

These findings suggest that the elevated T2_H2O_ and T1 values observed in young dogs reflect primarily the smaller cellular volume fraction and lower protein content typical of developing muscle tissue, while in GRMD, these abnormal values are likely to represent extracellular edema. This aligns with previous studies exploring ^31^P NMR spectroscopy, which suggest the increased relative fraction of a more alkaline inorganic phosphate pool observed in the skeletal muscle of GRMD dogs,^40^ DMD,^8^ and dysferlinopathy^7^ patients reflects an expanded interstitial space.

It is worth commenting on some discrepancies observed between ECV and A_2_. ECV was consistently higher than A_2_ across all age ranges (see Table 2). Notably, while both ECV and A_2_ decreased during the first year in both groups, ECV had a higher initial value, but a similar rate of decline compared to A_2_ (see Table 3), resulting in an increasing relative difference between these metrics. Given that ECV has been shown to correlate with fibrosis in animal models,^18^ including its use in assessing diffuse fibrosis in the myocardium,^15^, ^16^ and that diffuse fibrosis has been shown to have negligible impact on water T2 relaxation,^18^ the increasing relative difference between ECV and A_2_ with age could thus indicate the accumulation of fibrotic tissue, which predominantly affects ECV while having little influence on the T2 signal. Furthermore, it is likely that in injured and necrotizing muscle fibers, the sarcolemma becomes permeable to the Gd‐based contrast agent, thus resulting in increased measures of ECV, while having lower impact on A_2_.

It is important to acknowledge potential sources of bias in the metrics derived from our methodological approaches. First, the combined results from localized CPMG and T1 measurements strongly indicate that the long‐T2 component is characterized by a higher T1 compared to the main‐T2 component (see Tables 2 and 3). This would result in a weaker contribution of the long‐T2 component in the MSME signal acquired using a TR of 3 s. Nonetheless, our results suggest that such an effect must be rather small. Unlike T2_1_, both T2_H2O_ and A_2_ allowed differentiating GRMD from control dogs at all age ranges, showing a significant decrease with age in both groups, and a significantly faster decrease in GRMD dogs. Second, the fat‐suppression module used in the localized CPMG technique may produce magnetization transfer effects, which could cause a systematic bias on A_2_ if these effects differed between T2 components. Despite this, due to the nonnegligible fraction of intramuscular fat in healthy and diseased tissue, the multi‐exponential behavior of fat T2‐relaxation, and the inherent ill‐posed nature of multi‐exponential deconvolution, it is mandatory to exclude fat signal contamination to assess tissue water compartmentation via T2‐relaxometry. While we cannot rule out the possibility of such a bias, our results demonstrate the sensitivity of the metric A_2_ to pathophysiological processes involving changes in tissue water compartmentation.

In face of the ongoing research on therapeutic development for NMDs, either exploring gene therapies or targeting specific pathogenic pathways, there is an urgent need for more specific noninvasive biomarkers for clinical trials. In brain applications, multicomponent water T2 metrics have demonstrated their importance, offering a histologically validated measure of myelin content.^41^, ^42^, ^43^ Recently, myelin water fraction has been proposed as a biomarker for clinical trials looking for remyelination.^44^ Our results encourage further investigation into the multiexponential behavior of the water T2 relaxation in skeletal muscle, specifically within the context of NMDs, as this approach could provide comparable insights and benefits.

In conclusion, the qMRI/S metrics explored in this study provided highly sensitive markers for tracking disease activity in GRMD dogs. Structural and biochemical changes in the developing skeletal muscle significantly influence these metrics and should be carefully considered when evaluating the disease progression and treatment effects in GRMD. Notably, insights from ECV and bicomponent water T2 relaxometry suggest that (I) the elevated T1 and T2_H2O_ values observed in GRMD dogs are primarily due to the extracellular edema resulting from inflammatory responses to tissue injury and (II) the significant decrease in T1 and T2_H2O_ values with age in control and GRMD dogs is likely to reflect the progressive increase in cellular volume fraction and protein content associated with tissue growth.

Despite the relatively small sample size, our study underscores the potential of multicomponent water T2 relaxometry to provide valuable insights into muscle pathology in NMDs. Future research should focus on integrating histological data and expanding sample sizes to validate these findings further. The development of specific noninvasive biomarkers, as demonstrated in brain studies, could significantly improve clinical trials for NMD therapies, offering precise measures of disease activity and therapeutic efficacy.

## ETHICS STATEMENT

The study was conducted in conformity with the European regulatory laws concerning the use of animals for research and by the French Ministry of research (Ministère de l'Enseignement Supérieur et de la Recherche) under the following approval numbers: #3678, #23763, #36911, and 20/12/12‐18.

## Supporting information


**Figure S1:** Longitudinal histological assessment of the tibialis cranialis muscle in GRMD and control dogs.
Upper panel: Images of H&E‐stained tibialis cranialis muscle biopsies at x40 magnification, in healthy (upper line) and GRMD dogs (bottom line) at 2, 6 and 12 months of age (from left to right). The growth of the myofibers is obvious, as well as the consequent decrease of the extracellular space fraction in growing dogs.
Bottom panel: Data of a quantitative analysis performed on H&E‐stained tibialis cranialis muscle sections, according to a previously described method^1^, are presented. These results show that the volume fraction of endomysial and perimysial connective tissue (left graph) tends to decrease in both control and GRMD dogs between 2 months and the subsequent timepoints, while being elevated in GRMD dogs. Adiposis deposition (middle graph) barely develops in GRMD tibialis cranialis muscle, except in some more severely affected dogs at the age of 6 months and at low levels. Mononuclear cell infiltrates (right graph) is mainly found at early timepoints (2 and 6 months of age) and decreases thereafter (12 months).
1. Barthélémy, I. et al. Effects of an Immunosuppressive Treatment in the GRMD Dog Model of Duchenne Muscular Dystrophy. PLoS ONE 7, e48478 (2012).

## Data Availability

The data that support the findings of this study are available from the corresponding author upon reasonable request.
