# Supplementary figures and images for: Comprehensive quantitative magnetic resonance imaging assessment of skeletal muscle pathophysiology in golden retriever muscular dystrophy: Insights from multicomponent water T2 and extracellular volume fraction
Source: NMR Biomed. 2024 Oct 21;38(1):e5278. doi: 10.1002/nbm.5278 (PMC11602680; doi:10.1002/nbm.5278)

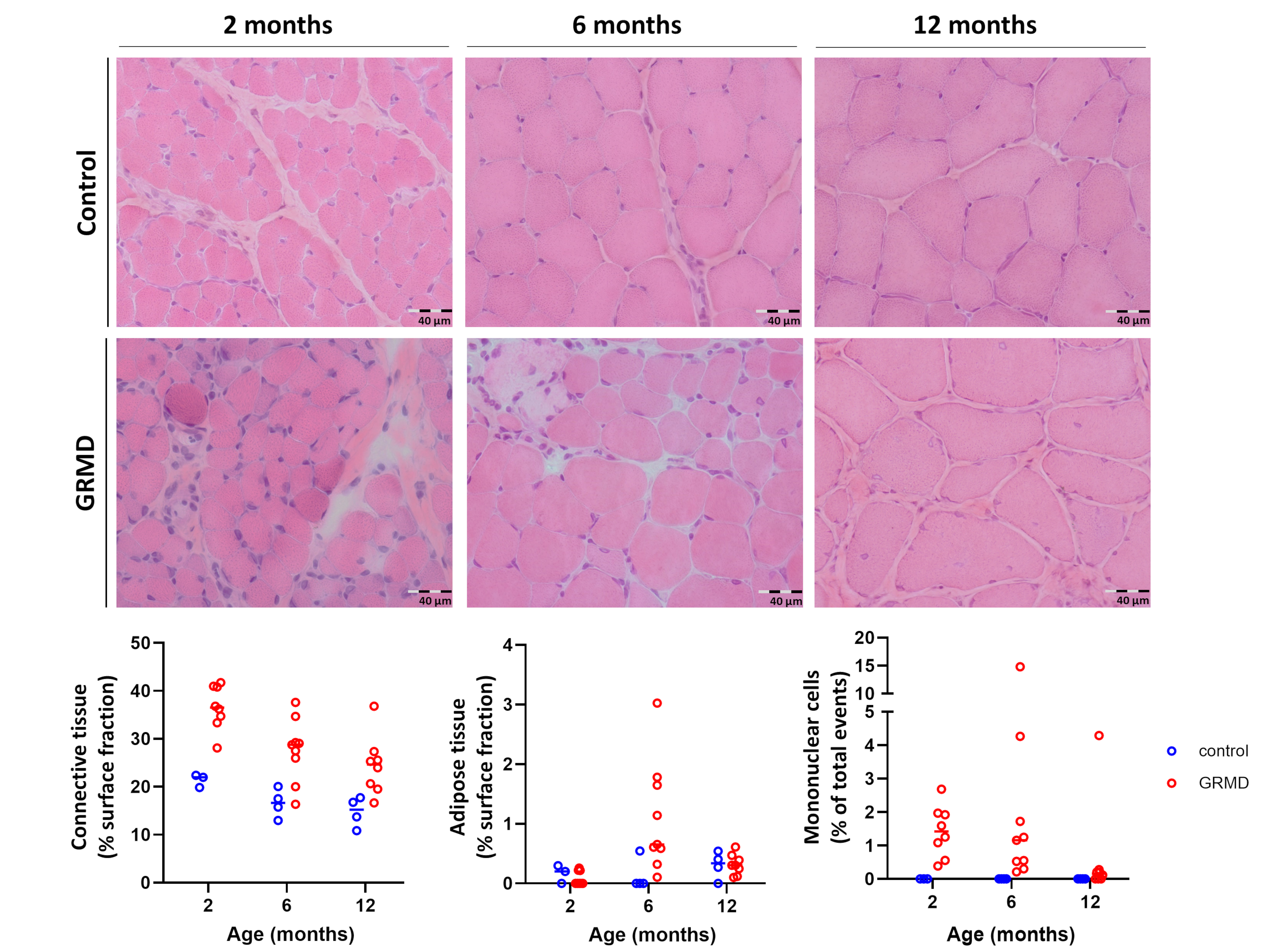

Supplement: Supplementary file 1 — Figure S1: Longitudinal histological assessment of the tibialis cranialis muscle in GRMD and control dogs. Upper panel: Images of H&E‐stained tibialis cranialis muscle biopsies at x40 magnification, in healthy (upper line) and GRMD dogs (bottom line) at 2, 6 and 12 months of age (from left to right). The growth of the myofibers is obvious, as well as the consequent decrease of the extracellular space fraction in growing dogs. Bottom panel: Data of a quantitative analysis performed on H&E‐stained tibialis cranialis muscle sections, according to a previously described method1, are presented. These results show that the volume fraction of endomysial and perimysial connective tissue (left graph) tends to decrease in both control and GRMD dogs between 2 months and the subsequent timepoints, while being elevated in GRMD dogs. Adiposis deposition (middle graph) barely develops in GRMD tibialis cranialis muscle, except in some more severely affected dogs at the age of 6 months and at low levels. Mononuclear cell infiltrates (right graph) is mainly found at early timepoints (2 and 6 months of age) and decreases thereafter (12 months). 1. Barthélémy, I. et al. Effects of an Immunosuppressive Treatment in the GRMD Dog Model of Duchenne Muscular Dystrophy. PLoS ONE 7, e48478 (2012). [file NBM-38-e5278-s001.tif]
